# Epidemiology and outcomes of multidrug-resistant bacterial infection in non-cystic fibrosis bronchiectasis

**DOI:** 10.1186/s12941-024-00675-6

**Published:** 2024-02-13

**Authors:** Chih-Hao Chang, Chiung-Hsin Chang, Shih-Hao Huang, Chung-Shu Lee, Po-Chuan Ko, Chun-Yu Lin, Meng-Heng Hsieh, Yu-Tung Huang, Horng-Chyuan Lin, Li-Fu Li, Fu-Tsai Chung, Chun-Hua Wang, Hung-Yu Huang

**Affiliations:** 1 Department of Thoracic Medicine, New Taipei City Municipal TuCheng Hospital, Chang Gung Medical Foundation, New Taipei City, Taiwan; 2 College of Medicine, Chang Gung University, Taoyuan, Taiwan; 3 Department of Thoracic Medicine, Chang Gung Memorial Hospital, 199 Tun-Hwa North Road, Taipei, Taiwan; 4 Center for Big Data Analytics and Statistics, Chang Gung Memorial Hospital, Taoyuan, Taiwan; 5 Division of Pulmonary and Critical Care Medicine, Department of Internal Medicine, Chang Gung Memorial Hospital, Keelung, Taiwan

**Keywords:** Bronchiectasis, Multidrug-resistant bacteria, Respiratory failure, Mortality

## Abstract

**Purpose:**

Multidrug-resistant (MDR) bacteria impose a considerable health-care burden and are associated with bronchiectasis exacerbation. This study investigated the clinical outcomes of adult patients with bronchiectasis following MDR bacterial infection.

**Methods:**

From the Chang Gung Research Database, we identified patients with bronchiectasis and MDR bacterial infection from 2008 to 2017. The control group comprised patients with bronchiectasis who did not have MDR bacterial infection and were propensity-score matched at a 1:2 ratio. The main outcomes were in-hospital and 3-year mortality.

**Results:**

In total, 554 patients with both bronchiectasis and MDR bacterial infection were identified. The types of MDR bacteria that most commonly affected the patients were MDR- *Acinetobacter baumannii* (38.6%) and methicillin-resistant *Staphylococcus aureus* (18.4%), Extended-spectrum-beta-lactamases (ESBL)- *Klebsiella pneumoniae* (17.8%), MDR-*Pseudomonas* (14.8%), and ESBL-*E. coli* (7.5%). Compared with the control group, the MDR group exhibited lower body mass index scores, higher rate of chronic bacterial colonization, a higher rate of previous exacerbations, and an increased use of antibiotics. Furthermore, the MDR group exhibited a higher rate of respiratory failure during hospitalization (MDR vs. control, 41.3% vs. 12.4%; *p* < 0.001). The MDR and control groups exhibited in-hospital mortality rates of 26.7% and 7.6%, respectively (*p* < 0.001); 3-year respiratory failure rates of 33.5% and 13.5%, respectively (*p* < 0.001); and 3-year mortality rates of 73.3% and 41.5%, respectively (*p* < 0.001). After adjustments were made for confounding factors, the infection with MDR and MDR bacteria species were determined to be independent risk factors affecting in-hospital and 3-year mortality.

**Conclusions:**

MDR bacteria were discovered in patients with more severe bronchiectasis and were independently associated with an increased risk of in-hospital and 3-year mortality. Given our findings, we recommend that clinicians identify patients at risk of MDR bacterial infection and follow the principle of antimicrobial stewardship to prevent the emergence of resistant bacteria among patients with bronchiectasis.

**Supplementary Information:**

The online version contains supplementary material available at 10.1186/s12941-024-00675-6.

## Introduction

Bronchiectasis is characterized by the permanent dilation of bronchi, recurrent respiratory infections and exacerbations [1]. Frequent exacerbations are associated with worse outcomes [2]. The main pathogens that cause exacerbations include bacteria, fungi, nontuberculous mycobacteria and viruses [3–5]. Bacterial infections can also occur during stable periods. Because bacterial colonization increases the risk of mortality in patients with bronchiectasis, it is included in bronchiectasis severity index (BSI) as a clinical prediction tool [6].

The incidence of multidrug-resistant (MDR) bacteria is increasing worldwide and becomes a threat to public health [7, 8]. Hospitalized patients, especially patients with critical illnesses and multimorbidity, are more likely to develop MDR bacterial infections because they undergo extensive antibiotic therapy [9]. Approximately 10–38% of hospitalized patients develop MDR bacterial infections during their hospital stay, and MDR bacterial infection is associated with increased hospital expenses, prolonged hospital stays, and higher mortality rates [9, 10].

Patients with bronchiectasis often receive antibiotics during exacerbations. The risk of acquiring MDR infection increases after several courses of broad-spectrum antibiotics treatment [11, 12]. In bronchiectasis, several risk factors for MDR infection were identified during exacerbations, including previous hospitalization, chronic kidney disease, and previous MDR isolation [11]. Increased risk of mortality has been implicated in patients with bronchiectasis and MDR infection but remain less well investigated [12]. In the international multicenter study which derives and validates BSI, methicillin-resistant *Staphylococcus aureus* (MRSA) infection and *Pseudomonas aeruginosa* infection exhibited the highest mortality rates (MRSA, 62.5%; *P. aeruginosa*, 21.2%) [6]. Because previous studies have had limited numbers of patients with both bronchiectasis and MDR bacterial infection, these studies have not analyzed the impact of each MDR bacterial species on clinical outcomes.

Few clinical studies have investigated the clinical outcomes of or identified the independent prognostic factors associated with MDR bacterial infection in patients with bronchiectasis. Even fewer studies have explored whether MDR bacterial species, patient characteristics, prior use of antibiotics, or comorbidities affect the clinical outcomes of bronchiectasis. To address this research gap, the present study investigated the risk factors associated with MDR bacterial infection in bronchiectasis and their effects on clinical outcomes in an Asian cohort.

## Methods

### Bronchiectasis cohort

This study analyzed data of Chang Gung Research Database (CGRD) from the electronic medical records of three medical centers and four regional hospitals that operate under in the Chang Gung Memorial Hospital (CGMH) system [13]. The multi-institutional bronchiectasis cohort comprised adult patients (aged ≥ 18 years) with at least two bronchiectasis diagnoses (*International Classification of Diseases, 9th Clinical Modification* (*ICD-9-CM*) 494.0 or 494.1) from outpatient visits or from hospitalization records from January 2008 to December 2017 [14–16]. The diagnosis of bronchiectasis was made by radiologist and pulmonary specialist based on the high-resolution computed tomography results and clinical symptoms. The exclusion criteria included patients without sputum culture, duration of follow up less than 6 months and cystic fibrosis.

### Definition of MDR infection

MDR bacteria were classified according to the international guideline [17]. For *Pseudomonas aeruginosa* and *Acinetobacter baumannii (AB)*, MDR was defined as resistance to at least three antimicrobial classes. For *S. aureus*, MRSA was defined as resistance to oxacillin. For *Escherichia coli (E. coli)* and *Klebsiella pneumoniae (KP)*, extended-spectrum-beta-lactamases (ESBL) was defined as resistance to at least three classes of β -lactam antibiotics, including penicillin, cephalosporins, and aztreonam. At least one culture of MDR pathogen was needed to define an individual as having MDR bacteria. The laboratory in CGMH used disc diffusion method (BD BBL Sensi-Disc, USA) to determine the susceptibility/resistance of antibiotics during the study period. The standard criteria of antibiotics sensitivity by disc diffusion method are added in the supplement Table S1. Chronic bacterial colonization which is defined as isolation of the same bacteria in two or more sputum cultures, at least 3 months apart within one year [18]. Bacterial infection in bronchiectasis was defined as an exacerbation related to bacteria with the use of antibiotics and worsening respiratory symptoms (increasing cough, sputum, or dyspnea) [19]. Convert to non-MDR status was defined as no growth of MDR bacteria in all sputum culture within one year. Because this study was based on a database of real-world practice, we did not set a minimum number of samples to define convert to non-MDR status.

### Main outcomes

The primary outcomes were in-hospital mortality and 3-year overall mortality after the index date, which was defined as the date of sputum culture collection prescribed by clinicians for suspicion of bronchiectasis exacerbation. Acute respiratory failure was defined according to *ICD-9-CM* code 518.81 or 518.82 or *ICD-10-CM* code J96.0 with mechanical ventilator use [20].

### Clinical parameters

Demographic data, CT reports, laboratory and microbiology data and pulmonary function reports were retrieved from the CGRD. The Bronchiectasis Aetiology Comorbidity Index (BACI) scores of the bronchiectasis cohort were calculated on the basis of their documented diagnoses (ICD-9-CM and ICD-10*-CM*) of comorbidities from CGRD [21]. The etiology of bronchiectasis was determined on the basis of the definition in another study [14]. Age was calculated from the birth date to the index date. The clinical details (body mass index (BMI) and lung function) were retrieved from the medical records within one year before index date. Comorbidities were retrieved from the medical records within three years before index date. We retrieved sputum microbiology reports including bacteria species and antibiotics sensitivity. Shock was defined according to diagnoses (*ICD-9-CM* code 785 or ICD-10*-CM* code R57) involving the use of systemic inotropic agents or vasopressors [22]. The medical treatments considered in the present study included systemic antibiotics, inhalation antibiotics, systemic corticosteroids, and inhalation steroids. Acute kidney injury during hospital admission was defined on the basis of serum creatinine level (an increase of 0.3 mg/dL or of 50% above baseline) [23].

### Statistical analysis

Because an imbalance was identified in the distribution of clinical characteristics between the MDR and control groups, propensity score matching was performed to address potential confounding factors. The predicted probability of identifying positive MDR isolates was calculated through logistic regression and served as the propensity score, which incorporated several covariates related to outcomes, namely age, sex, comorbidities, and BACI score (Table 1). The MDR and control groups were matched at a 1:2 ratio.

**Table 1:** Demographics and clinical characteristics of patients

	Control group	MDR group	p-value	MDR-AB	ESBL-E coli	ESBL-KP	MDR-Pseudomonus	MRSA	p-value
	*n*=1108	*n*=554		*n*=214	*n*=47	*n*=99	*n*=82	*n*=102	
Age	75.0 ± 11.7	75.1 ± 12.7	0.968	74.5 ± 12.2	74.8 ± 12.9	77.1 ± 11.1	70.9 ± 13.3	77.1 ± 13.9	0.006
Sex (Female)	455 (41.1%)	228 (41.2%)	0.972	95 (44.4%)	16 (34.0%)	32 (32.3%)	43 (52.4%)	41 (40.2%)	0.053
BMI	22.34 ± 4.2	20.9 ± 4.3	<0.001	20.9 ± 4.3	21.2 ± 3.1	20.8 ± 4.4	20.3 ± 3.6	21.9 ± 5.0	0.181
Pulmonary function			<0.001						0.246
FVC: <80 pred.%	243 (33.3%)	150 (44.3%)		59 (45.0%)	19 (67.9%)	24 (48.0%)	26 (43.3%)	21 (33.3%)	
FEV1: > 80 pred.%	219 (30.0%)	58 (17.1%)		19 (14.5%)	3 (10.7%)	6 (12.0%)	10 (16.7%)	18 (28.6%)	
FEV1: 50–80 pred.%	154 (21.1%)	53 (15.6%)		21 (16.0%)	3 (10.7%)	8 (16.0%)	11 (18.3%)	10 (15.9%)	
FEV1: <50 pred.%	114 (15.6%)	78 (23.0%)		32 (24.4%)	3 (10.7%)	12 (24.0%)	13 (21.7%)	14 (22.2%)	
Previous exacerbation*	2.3 ± 6.6	3.9 ± 6.6	<0.001	3.7 ± 4.0	5.6 ± 12.9	3.5 ± 3.3	3.7 ± 3.3	4.3 ± 10.3	0.419
0	284 (25.6%)	40 (7.2%)	<0.001	16 (7.5%)	3 (6.4%)	5 (5.1%)	7 (8.5%)	9 (8.8%)	0.811
1	408 (36.8%)	142 (25.6%)		57 (26.6%)	11 (23.4%)	21 (21.2%)	20 (24.4%)	30 (29.4%)	
2	158 (14.3%)	90 (16.3%)		29 (13.6%)	9 (19.2%)	23 (23.2%)	12 (14.6%)	16 (15.7%)	
≥ 3	258 (23.3%)	282 (50.9%)		112 (52.3%)	24 (51.1%)	50 (50.5%)	43 (52.4%)	47 (46.1%)	
BACI index	11.0 ± 6.8	11.3 ± 6.4	0.358	10.4 ± 5.8	12.3 ± 8.5	11.0 ± 5.9	11.9 ± 6.4	12.5 ± 6.9	0.047
Comorbidity									
Solid tumor	126 (11.4%)	64 (11.6%)	0.913	20 (9.4%)	10 (21.3%)	8 (8.1%)	10 (12.2%)	16 (15.7%)	0.087
Hematological malignancy	56 (5.1%)	25 (4.5%)	0.629	10 (4.7%)	1 (2.1%)	2 (2.0%)	6 (7.3%)	6 (5.9%)	0.436
COPD	737 (66.5%)	365 (65.9%)	0.797	133 (62.2%)	29 (61.7%)	64 (64.7%)	62 (75.6%)	70 (68.6%)	0.236
Liver disease	308 (27.8%)	143 (25.8%)	0.391	44 (20.6%)	12 (25.5%)	26 (26.3%)	27 (32.9%)	30 (29.4%)	0.201
Connective tissue disease	84 (7.6%)	39 (7.0%)	0.691	13 (6.1%)	1 (2.1%)	6 (6.1%)	8 (9.8%)	11 (10.8%)	0.268
Diabetes	452 (40.8%)	226 (40.8%)	1	80 (37.4%)	24 (51.1%)	43 (43.4%)	30 (36.6%)	43 (42.2%)	0.402
Asthma	500 (45.1%)	256 (46.2%)	0.676	110 (51.4%)	22 (46.8%)	41 (41.4%)	38 (46.3%)	42 (41.2%)	0.372
Ischemic heart disease	348 (31.4%)	151 (27.3%)	0.082	58 (27.1%)	11 (23.4%)	23 (23.2%)	22 (26.8%)	35 (34.3%)	0.446
Cerebrovascular disease	418 (37.7%)	206 (37.2%)	0.829	67 (31.3%)	17 (36.2%)	48 (48.5%)	21 (25.6%)	49 (48.0%)	0.001
Cardiovascular disease	586 (52.9%)	285 (51.4%)	0.578	99 (46.3%)	23 (48.9%)	60 (60.6%)	34 (41.5%)	64 (62.8%)	0.007
Chronic renal disease	363 (32.8%)	176 (31.8%)	0.684	56 (26.2%)	15 (31.9%)	38 (38.4%)	24 (29.3%)	41 (40.2%)	0.071
GERD	359 (32.4%)	176 (31.8%)	0.795	63 (29.4%)	13 (27.7%)	21 (21.2%)	31 (37.8%)	42 (41.2%)	0.021
Osteoporosis	272 (24.6%)	141 (25.5%)	0.688	52 (24.3%)	10 (21.3%)	21 (21.2%)	24 (29.3%)	31 (30.4%)	0.483
CGMH institutes			<0.001						0.002
Keelung	182 (16.4%)	118 (21.3%)		51 (23.8%)	11 (23.4%)	15 (15.2%)	9 (10.9%)	31 (30.4%)	
Linkou and Taipei	392 (35.4%)	236 (42.6%)		85 (39.7%)	25 (53.2%)	47 (47.5%)	40 (48.8%)	34 (33.3%)	
Chiayi	256 (23.1%)	128 (23.1%)		39 (18.2%)	7 (14.9%)	27 (27.3%)	20 (24.4%)	31 (30.4%)	
Kaohsiung	245 (22.1%)	70 (12.6%)		38 (17.8%)	3 (6.4%)	10 (10.1%)	13 (15.9%)	6 (5.9%)	
Yunlin	33 (2.9%)	2 (0.4%)		1 (0.5%)	1 (2.1%)	0 (0%)	0 (0%)	0 (0%)	

*Note* AB, Acinetobacter baumannii; BACI, bronchiectasis aetiology comorbidity index; BMI: body mass index; CGMH, Chang Gung Memorial Hospital; COPD, chronic obstruction pulmonary disease; ESBL, extended-spectrum-beta-lactamases; FEV1, forced expiratory volume in one second; FVC, forced vital capacity; GERD, Gastroesophageal reflux disease; KP, Klebsiella pneumoniae; MDR: multidrug- resistant; pred.; MRSA, methicillin-resistant Staphylococcus aureus; predicted value; * Previous exacerbation within one year

For dichotomous variables, we used chi-square tests and two-sided Fisher exact tests for analysis. Unpaired t-tests were used for normally distributed continuous variables. For non-normally distributed continuous data, Mann-Whitney U tests were used for analysis. P-values (two-sided) < 0.05 were considered statistically significant. Risk factors for mortality were identified through univariate descriptive analysis. For the variables with significant results (*p* < 0.05, as revealed through univariate descriptive analysis), multivariate Cox proportional-hazards regression was performed to identify independent risk factors. Statistical analyses were performed using SAS software, version 9.4 (SAS Institute, Cary, North Carolina, USA).

## Results

From the CGRD, 8,063 patients with bronchiectasis and sputum culture were identified between 2008 and 2017. A total of 554 bronchiectasis patients having MDR infection were confirmed. After propensity score matching was performed, the MDR and control groups exhibited similar distributions for age, sex, and comorbidities (supplementary Table S2). In the MDR group, 214 (38.6%) were MDR-*AB*, 102 (18.4%) were MRSA, 99 (17.8%) were ESBL-*KP*, 82 (14.8%) were MDR-*Pseudomonas*, and 47 (7.5%) were *ESBL-E. coli*. The distribution of patients with bronchiectasis in the CGMH-affiliated institutions is presented in Table 1, which reveals that the Linkou branch accounted for the largest proportion of the patients with MDR (42.6%), followed by the Chiayi branch (23.1%) and Keelung branch (21.3%). The bacteriological results were mainly from sputum culture (MDR:95.4%, control: 94.9%). Only a small portion of bacteriological results were from bronchial washing or bronchoalveolar larvage culture reports (MDR:4.6%, control: 5.1%).

**Fig. 1:**
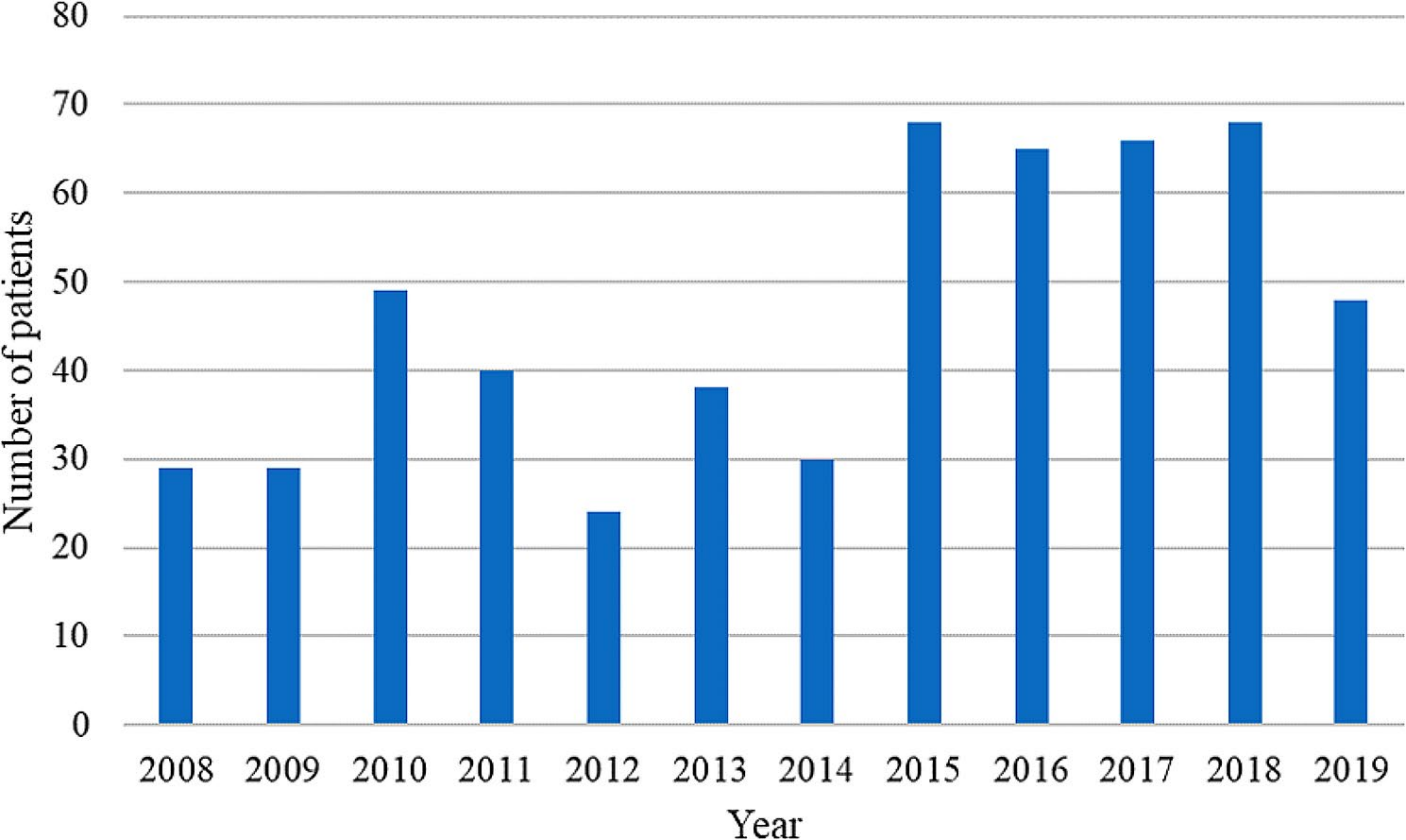
Annual incident numbers of bronchiectasis with multidrug-resistant bacterial infection in CGRD since 2008–2017. CGRD: Chang Gung Research Database

**Fig. 2:**
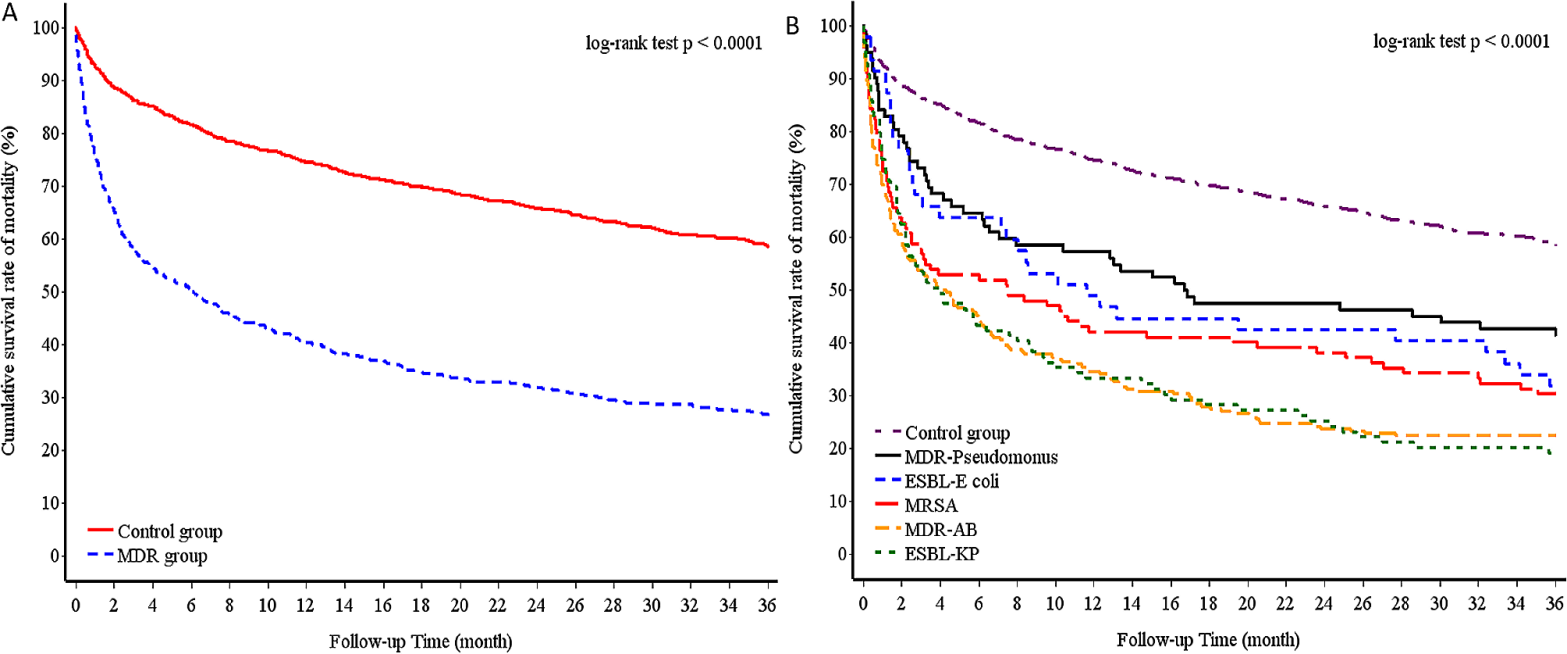
Kaplan–Meier survival curves for (**A**) 3-year mortality of the cohort (Control and MDR groups); (**B**) 3-year mortality of the cohort (Control and MDR subgroups) MDR, multidrug-resistant

The demographic and clinical characteristics are summarized in Table 1. Compared with the control group, the MDR group exhibited lower FEV1 levels and BMI scores, a higher rate of previous exacerbation, and an increased use of antibiotics. The MDR group had a significantly higher rate of chronic bacterial colonization than control group (90 (16.25%) vs. 17 (1.53%), *p* < 0.001). During treatment, the MDR group exhibited higher rates of acute kidney injury and hemodialysis than the control group. Relative to the patients in the control group, a higher proportion of the patients in the MDR group were administered antibiotics (MDR vs. control, 98.2% vs. 79.9%; *p* < 0.001) and systemic corticosteroids (MDR vs. control, 71.3% vs. 42.5%; *p* < 0.001) (Table 2). 130 patients (MDR group: 40) had non-tuberculous mycobacterium in sputum culture within one months of index date. 37 patients (MDR group: 21) had mycobacterium avium-intracellulare complex in sputum culture. 1164 patients (MDR group: 517) had blood culture and 240 patients (MDR group: 151) had positive pathogen results in blood culture. 28 patients had MDR bacteria in blood culture and were all in the MDR group. The MDR group exhibited a higher rate of respiratory failure during hospitalization (MDR vs. control, 41.3% vs. 12.4%; *p* < 0.001) (Table 3). The MDR and control groups had an in-hospital mortality rate of 26.7% and 7.6%, respectively (*p* < 0.001); a 3-year respiratory failure rate of 33.6% and 13.5%, respectively (*p* < 0.001); and a 3-year mortality rate of 73.3% and 41.5%, respectively (*p* < 0.001) (Table 3). Respiratory failure (MDR: 90(60.8%), control: 56(67.5%)) was the major cause of in-hospital mortality and other causes included cardiovascular deaths (MDR:14(9.4%), control: 10(12.0%)) and other deaths (MDR:44(29.7%), control: 17(20.5%)). Respiratory failure (MDR:255(62.8%), control: 271(58.9%)) was the major cause of 3-year mortality and other causes included cardiovascular deaths (MDR:35(8.6%), control: 70(15.2%)) and other deaths (MDR:116(28.6%), control: 119(25.9%)). In MDR group, 42.1% convert to non-MDR status during follow up. The mean number of negative sputum culture in convert to non-MDR patients were 4.95 ± 4.51 within one year and 83.2% of them had more than two times of negative sputum culture. Relative to the control group, the MDR group had a considerably higher cumulative incidence of mortality during a 3-year follow-up period (Fig. 1a). The mean duration of follow up was 2.1 ± 0.8 years for MDR group and 2.7 ± 1.3 years for control group (*p* < 0.01). Figure 2 presents the incidence of MDR infection stratified by year.

**Table 2:** Antibiotics exposure, clinical parameters and treatment during hospitalization

	Control group	MDR group	p-value	MDR-AB	ESBL-E coli	ESBL-KP	MDR-Pseudomonus	MRSA	p-value
	*n*=1108	*n*=554		*n*=214	*n*=47	*n*=99	*n*=82	*n*=102	
Previous antibiotics*									
Penicillin/ b -lactamase inhibitor	416 (37.55%)	261 (47.11%)	0.001	107 (50%)	25 (53.19%)	44 (44.44%)	36 (43.9%)	45 (44.12%)	0.663
Antipseudomonal penicillins	92 (8.3%)	299 (53.97%)	<0.001	123 (57.48%)	21 (44.68%)	60 (60.61%)	35 (42.68%)	53 (51.96%)	0.066
1st or 2nd generation cephalosporin	367 (33.12%)	242 (43.68%)	<0.001	87 (40.65%)	27 (57.45%)	49 (49.49%)	33 (40.24%)	39 (38.24%)	0.118
3th or 4th generation cephalosporin	321 (28.97%)	445 (80.32%)	<0.001	175 (81.78%)	35 (74.47%)	83 (83.84%)	62 (75.61%)	80 (78.43%)	0.498
Carbapenems	42 (3.79%)	256 (46.21%)	<0.001	108 (50.47%)	11 (23.4%)	56 (56.57%)	47 (57.32%)	30 (29.41%)	<0.001
Fluoroquinolones	487 (43.95%)	418 (75.45%)	<0.001	172 (80.37%)	35 (74.47%)	73 (73.74%)	61 (74.39%)	69 (67.65%)	0.173
Antibiotics in ward									
Antibiotics days	9.1 ± 9.0	14.9 ± 11.5	<0.001	15.6 ± 10.9	14.5 ± 13.7	16.6 ± 11.8	14.7 ± 13.9	12.2 ± 9.1	0.105
Penicillin/ b -lactamase inhibitor	240 (21.7%)	86 (15.5%)	0.003	52 (24.3%)	6 (12.8%)	9 (9.1%)	11 (13.4%)	6 (5.9%)	<0.001
Antipseudomonal penicillins	95 (8.6%)	121 (21.8%)	<0.001	38 (17.8%)	12 (25.5%)	21 (21.2%)	20 (24.4%)	26 (25.5%)	0.463
1st or 2nd generation cephalosporin	206 (18.6%)	36 (6.5%)	<0.001	11 (5.1%)	6 (12.8%)	7 (7.1%)	3 (3.7%)	9 (8.8%)	0.229
3th or 4th generation cephalosporin	252 (22.7%)	238 (42.9%)	<0.001	86 (40.2%)	19 (40.4%)	39 (39.4%)	36 (43.9%)	54 (52.9%)	0.241
Carbapenems	60 (5.4%)	219 (39.5%)	<0.001	84 (39.3%)	19 (40.4%)	57 (57.6%)	27 (32.9%)	27 (26.5%)	0.001
Fluoroquinolones	345 (31.1%)	182 (32.9%)	0.479	57 (26.6%)	14 (29.8%)	41 (41.4%)	35 (42.7%)	30 (29.4%)	0.022
Glycopeptide	85 (7.7%)	262 (47.3%)	<0.001	111 (51.9%)	7 (14.9%)	40 (40.4%)	23 (28.1%)	77 (75.5%)	<0.001
Aminoglycoside	11 (0.9%)	33 (5.9%)	<0.001	10 (4.7%)	1 (2.1%)	8 (8.1%)	7 (8.5%)	5 (4.9%)	0.416
Other	159 (14.4%)	242 (43.7%)	<0.001	143 (66.8%)	11 (23.4%)	28 (28.3%)	34 (41.5%)	24 (23.5%)	<0.001
Time from diagnosis (months)	5.2 ± 17.2	28.1 ± 30.9	<0.001	22.9 ± 27.9	16.1 ± 17.5	20.8 ± 25.5	38.9 ± 33.6	41.9 ± 35.6	<0.001
WBC (×10^3 /uL)	10.4 ± 7.3	13.1 ± 14.1	<0.001	12.9 ± 6.5	9.9 ± 6.4	13.0 ± 5.9	12.1 ± 5.0	15.9 ± 30.3	0.157
Platelet (×10^3 /uL)	226.5 ± 92.1	229.1 ± 106.8	0.629	241.8 ± 112.5	201.3 ± 98.6	213.8 ± 95.1	230.7 ± 93.6	229.5 ± 116.6	0.085
C-reactive protein (mg/L)	64.8 ± 74.5	79.9 ± 84.2	0.002	75.8 ± 75.3	63.5 ± 78.6	103.9 ± 102.5	70.4 ± 80.3	81.4 ± 89.3	0.055
Creatinine,baseline (mg/dL)	1.3 ± 1.3	1.4 ± 1.5	0.533	1.3 ± 1.6	1.5 ± 1.3	1.5 ± 1.6	0.9 ± 0.9	1.6 ± 1.6	0.019
Creatinine,ward (mg/dL)	1.4 ± 1.6	1.8 ± 1.9	0.001	1.8 ± 1.9	1.8 ± 1.8	1.9 ± 1.9	1.4 ± 1.4	1.9 ± 2.0	0.354
Acute kidney injury	149 (13.5%)	157 (28.3%)	<0.001	69 (32.2%)	12 (25.5%)	31 (31.3%)	16 (19.5%)	24 (23.5%)	0.162
Hemodialysis	34 (3.1%)	60 (10.8%)	<0.001	22 (10.3%)	8 (17.0%)	15 (15.2%)	4 (4.9%)	10 (9.8%)	0.138
Inhospital medication									
Systemic steroid	471 (42.5%)	395 (71.3%)	<0.001	165 (77.1%)	35 (74.5%)	66 (66.7%)	50 (60.9%)	71 (69.6%)	0.057
Inhalation steroid	135 (12.2%)	118 (21.3%)	<0.001	53 (24.8%)	9 (19.2%)	23 (23.2%)	21 (25.6%)	11 (10.8%)	0.051
Antibiotic	885 (79.9%)	544 (98.2%)	<0.001	211 (98.6%)	46 (97.9%)	98 (98.9%)	79 (96.3%)	100 (98.0%)	0.662
Inhalation gentamicin	51 (4.6%)	50 (9.0%)	0.001	22 (10.3%)	4 (8.5%)	6 (6.1%)	11 (13.4%)	7 (6.9%)	0.419

*Note* AB, Acinetobacter baumannii; ESBL, extended-spectrum-beta-lactamases; KP, Klebsiella pneumoniae; MDR: multidrug- resistant; MRSA, methicillin-resistant Staphylococcus aureus; WBC, white blood cell count

* Previous antibiotics 6 months before index date

**Table 3:** Main clinical outcomes of hospitalization

	Control group	MDR group	p-value	MDR-AB	ESBL-E coli	ESBL-KP	MDR-Pseudomonus	MRSA	p-value
	*n*=1108	*n*=554		*n*=214	*n*=47	*n*=99	*n*=82	*n*=102	
Length of ward (days)	12.6 ± 10.5	26.4 ± 22.3	<0.001	27.3 ± 16.8	29.9 ± 20.5	27.3 ± 17.5	28.6 ± 42.2	20.3 ± 12.8	0.043
Length of ICU (days)	9.8 ± 9.2	18.9 ± 14.2	<0.001	19.3 ± 12.5	17.2 ± 16.9	20.0 ± 14.6	22.4 ± 18.5	13.7 ± 12.6	0.068
Respiratory failure	137 (12.4%)	229 (41.3%)	<0.001	97 (45.3%)	11 (23.4%)	41 (41.4%)	36 (43.9%)	41 (40.2%)	0.096
Invasive MV	77 (6.9%)	150 (27.1%)	<0.001	69 (32.2%)	7 (14.9%)	28 (28.3%)	26 (31.7%)	19 (18.6%)	0.027
Non-invasive MV	89 (8.0%)	125 (22.6%)	<0.001	42 (19.6%)	7 (14.9%)	22 (22.2%)	21 (25.6%)	31 (30.4%)	0.156
Duration of ventilator (days)	7.8 ± 7.4	17.9 ± 13.7	<0.001	18.2 ± 12.8	17.1 ± 13.6	18.5 ± 13.9	21.6 ± 18.2	11.7 ± 9.1	0.033
Shock	67 (6.1%)	169 (30.5%)	<0.001	68 (31.8%)	9 (19.2%)	40 (40.4%)	23 (28.1%)	25 (24.5%)	0.048
Inhospital mortality	83 (7.6%)	148 (26.7%)	<0.001	67 (31.3%)	10 (21.3%)	27 (27.3%)	15 (18.3%)	27 (26.5%)	0.201
Convert to non-MDR	(%)	233 (42.1%)		88 (41.1%)	24 (51.1%)	39 (39.4%)	31 (37.8%)	45 (44.1%)	0.609
Time to non-MDR (days)	(%)	27.2 ± 64.8		15.1 ± 29.1	37.5 ± 52.6	28.9 ± 87.1	32.8 ± 71.9	47.3 ± 94.8	0.004
3 year Respiratory failure	150 (13.5%)	186 (33.6%)	<0.001	79 (36.9%)	11 (23.4%)	35 (35.4%)	35 (42.7%)	23 (22.6%)	<0.001
Invasive MV	101 (9.1%)	149 (26.9%)	<0.001	66 (30.8%)	7 (14.9%)	29 (29.3%)	32 (39.0%)	14 (13.7%)	<0.001
Non-invasive MV	87 (7.9%)	84 (15.2%)	<0.001	30 (14.0%)	6 (12.8%)	14 (14.1%)	13 (15.9%)	18 (17.7%)	0.001
3 year mortality	460 (41.5%)	406 (73.3%)	<0.001	166 (77.6%)	32 (68.1%)	80 (80.8%)	48 (58.5%)	71 (69.6%)	<0.001

*Note* AB, Acinetobacter baumannii; ESBL, extended-spectrum-beta-lactamases; KP, Klebsiella pneumoniae; MDR: multidrug- resistant; MRSA, methicillin-resistant Staphylococcus aureus; MV, mechanical ventilator

The characteristics of the MDR subgroups are presented in Table 1, which reveals that the MDR subgroups had similar distributions for sex, BMI score, lung function, and acute exacerbation rate in the preceding year. Among the MDR subgroups, the ESBL-*KP* subgroup had the highest mean age, and the MRSA subgroup had the highest BACI scores. Furthermore, the ESBL-*KP* and MRSA subgroups had higher proportions of patients with preexisting cerebrovascular disease and cardiovascular disease relative to the other subgroups.

The outcomes of the MDR subgroups are presented in Table 3. Among the MDR subgroups, the ESBL-*E. coli* subgroup exhibited the shortest time to MDR infection following a diagnosis of bronchiectasis, whereas the MRSA subgroup exhibited the longest time to MDR infection following a diagnosis of bronchiectasis. In addition, the MDR-*AB* subgroup had the highest rate of invasive ventilator use; the MDR-*Pseudomonas* subgroup had the highest number of days of ventilator use; the ESBL-*KP* subgroup had the highest incidence of shock (47% vs. 6%; *p* < 0.001); the ESBL-*E. coli* subgroup had the longest duration of hospital stay; the MDR-*Pseudomonas* subgroup had the highest 3-year respiratory failure rate; the ESBL-*KP* subgroup had the highest 3-year mortality rate; and the MDR-*AB* subgroup required the least time to convert from an MDR to a non-MDR status. For in-hospital mortality, the MDR subgroups did not exhibit significant differences. Figure 1b presents the survival curves of the MDR subgroups during a 3-year follow-up period.

Through multivariate analysis, several independent factors for in-hospital mortality were identified (Table 4), namely MDR bacteria (odds ratio [OR], 2.41; 95% confidence interval [CI], 1.80–3.23; *p* < 0.001), age (OR, 1.02; 95% CI, 1.01–1.03; *p* = 0.001), hematological malignancy (OR, 1.97; 95% CI, 1.21–3.21; *p* = 0.006), and acute kidney injury (OR, 3.02; 95% CI, 2.28–4.00; *p* < 0.001). The analysis also revealed several independent risk factors for 3-year mortality (Table 5), namely MDR bacteria (OR, 1.90; 95% CI, 1.65–2.19; *p* < 0.001), age (OR, 1.04; 95% CI, 1.03–1.04; *p* < 0.001), male sex (OR, 7.66; 95% CI, 3.73–15.75; *p* < 0.001), per-unit decrease in BMI score (OR, 7.66; 95% CI, 3.73–15.75; *p* < 0.001), acute kidney injury (OR, 7.66; 95% CI, 3.73–15.75; *p* < 0.001), BACI score (OR, 7.66; 95% CI, 3.73–15.75; *p* < 0.001), and hemodialysis (OR, 0.99; 95% CI, 0.99–0.99; *p* = 0.021). MDR conversion and MDR nonconversion were both independent risk factors for 3-year mortality after adjustments were made for confounding factors (Table 5). All MDR bacteria except ESBL-*E. coli* were independent risk factors for in-hospital and 3-year mortality in the bronchiectasis cohort after adjustments were made for confounding factors (Table 6).

**Table 4:** Univariate and multivariate analysis of in-hospital mortality

In-hospital mortality	Univariate Analysis		Multivariate Analysis	
Control group	Ref.		Ref.	
MDR group	3.079 (2.322-4.084)	<0.001	2.408 (1.795-3.231)	<0.001
Age	1.022 (1.010-1.035)	0.0010	1.020 (1.008-1.033)	0.001
BMI	0.966 (0.933-0.999)	0.044	0.991 (0.956-1.028)	0.633
WBC (×10^3 /uL)	1.016 (1.009-1.023)	<0.001	1.013 (1.002-1.025)	0.017
Platelet (×10^3 /uL)	0.998 (0.997-0.999)	0.019	0.999 (0.997-1.000)	0.052
C-reactive protein (mg/L)	1.003 (1.002-1.005)	<0.001	1.001 (1.000-1.003)	0.119
Acute kidney injury	4.161 (3.184-5.439)	<0.001	3.024 (2.284-4.004)	<0.001
Hematological malignancy	2.140 (1.363-3.358)	0.001	1.971 (1.209-3.212)	0.007
Diabetes mellitus	0.734 (0.556-0.969)	0.029	0.731 (0.551-0.970)	0.031
Asthma	0.619 (0.469-0.817)	0.001	0.685 (0.516-0.910)	0.009
GERD	0.546 (0.396-0.752)	0.001	0.727 (0.523-1.010)	0.057

*Note* BMI: body mass index; GERD: Gastroesophageal reflux disease; MDR: multidrug- resistant; WBC, white blood cell count

**Table 5:** Univariate and multivariate analysis of 3-year mortality

3-year mortality	Univariate Analysis		Multivariate Analysis model 1		Multivariate Analysis model 2	
Control group	Ref.		Ref.		Ref.	
MDR group	2.697 (2.357-3.085)	<0.001	1.900 (1.645-2.193)	<0.001		
Control group	Ref.				Ref.	
MDR conversion	2.711 (2.316-3.172)	<0.001			1.850 (1.566-2.186)	<0.001
MDR non-conversion	2.677 (2.241-3.198)	<0.001			1.971 (1.640-2.367)	<0.001
Age	1.039 (1.032-1.045)	<0.001	1.036 (1.029-1.043)	<0.001	1.036 (1.029-1.043)	<0.001
Sex (Female)	0.695 (0.605-0.799)	<0.001	0.745 (0.647-0.858)	<0.001	0.746 (0.648-0.858)	<0.001
BMI	0.934 (0.917-0.951)	<0.001	0.950 (0.933-0.967)	<0.001	0.950 (0.933-0.967)	<0.001
Previous exacerbation*	1.010 (1.004-1.016)	0.002	1.003 (0.995-1.102)	0.464	1.003 (0.995-1.012)	0.469
BACI index	1.036 (1.026-1.045)	<0.001	1.019 (1.009-1.030)	0.001	1.019 (1.009-1.030)	0.001
Acute kidney injury	2.329 (1.997-2.716)	<0.001	1.789 (1.526-2.098)	<0.001	1.793 (1.529-2.103)	<0.001
Hemodialysis	2.025 (1.583-2.590)	<0.001	1.593 (1.217-2.084)	0.001	1.602 (1.224-2.098)	0.001
Inhospital medication						
Systemic steroid	2.200 (1.914-2.529)	<0.001	1.643 (1.413-1.910)	<0.001	1.645 (1.415-1.913)	<0.001
Inhalation steroid	1.327 (1.115-1.579)	0.001	0.998 (0.834-1.194)	0.979	0.999 (0.835-1.195)	0.989
Antibiotic	3.707 (2.778-4.946)	<0.001	1.862 (1.376-2.519)	<0.001	1.859 (1.374-2.515)	<0.001

*Note* AB, Acinetobacter baumannii; BACI, bronchiectasis aetiology comorbidity index; BMI: body mass index; MDR: multidrug- resistant

**Table 6:** Adjusted hazard ratio of in-hospital and 3-year mortality in MDR subgroups

	In-hospital mortality		3-year mortality	
Control group	Ref.		Ref.	
MDR subgroups				
MDR-AB	2.865 (2.024-4.055)	<0.001	2.376 (1.971-2.864)	<0.001
ESBL-E coli	1.816 (0.898-3.672)	0.097	1.204 (0.833-1.741)	0.324
ESBL-KP	2.184 (1.371-3.480)	0.001	2.190 (1.715-2.796)	<0.001
MDR-Pseudomonus	1.856 (1.055-3.265)	0.032	1.525 (1.125-2.066)	0.007
MRSA	2.551 (1.594-4.082)	<0.001	1.611 (1.248-2.080)	0.001

*Note* AB, Acinetobacter baumannii; ESBL, extended-spectrum-beta-lactamases; KP, Klebsiella pneumoniae; MDR: multidrug- resistant; MRSA, methicillin-resistant Staphylococcus aureus

## Discussion

This study revealed an association between MDR bacterial infection and poor outcomes in patients with bronchiectasis. In our bronchiectasis cohort, MDR-*AB* was the most frequently isolated MDR bacteria, followed by MRSA, ESBL-*KP*, MDR-*Pseudomonas*, and ESBL-*E. coli*. Relative to the control group, the MDR group exhibited significantly higher rates of in-hospital mortality, 3-year respiratory failure, and 3-year mortality. All MDR subgroups (stratified by MDR bacteria species) exhibited a higher risk of mortality relative to the control group.

The prevalence of MDR bacteria is increasing worldwide. In the Unites States, more than 2 million patients were infected with MDR pathogens annually [24]. In Asia, high rates of MDR bacterial isolation have been reported in patients in normal wards and intensive care units (ICU) [7]. The substantial burden of antimicrobial MDR bacteria poses a public health problem and leads to increased morbidity, mortality, and medical expenses [7–9]. The prevalence of MDR bacteria varies across different countries. In a European study that analyzed a cohort of patients with bronchiectasis, MDR bacteria were isolated in 20% of exacerbation cases, with the most frequently isolated bacteria being *Pseudomonas*, MRSA, and ESBL- *Enterobacteriaceae* [11]. Studies have reported significant geographic variations in the prevalence of pathogens during bronchiectasis exacerbations [25]. However, the literature on the prevalence of MDR bacterial infection among patients with bronchiectasis in Asia is limited. In the bronchiectasis cohort of the present study (retrieved), the most frequently identified MDR bacteria were MDR-*AB* (38.6%), MRSA (18.4%), ESBL-*KP* (17.8%), MDR-*Pseudomonas* (14.8%), and ESBL-*E. coli* (7.5%). In Asia, the emergence and spread of MDR bacterial infection among hospitalized patients is becoming a health-care concern. To the best of our knowledge, the present study is the first to report on the characteristics and outcomes of MDR bacterial infection in patients with bronchiectasis in Asia.

The effects of MDR bacterial infection on outcomes is a key clinical topic that should be investigated. Nosocomial pneumonia due to MDR bacteria has been reported to be associated with poor clinical outcomes [9, 10]. MDR bacteria were independently associated with increased in-hospital, 1-month, and 6-month mortality in patients undergoing mechanical ventilation [9, 26]. MDR bacteria are commonly identified in patients with chronic obstructive pulmonary disease (COPD) who experience severe acute exacerbations that require intubation and mechanical ventilation [27, 28]. A study reported MDR infection caused higher mortality rate than drug-sensitive patients in COPD exacerbation (12%, vs. 4.7%) [29]. The effects of MDR bacterial infection on the clinical outcomes of patients with bronchiectasis require further clarification. Our findings indicate that MDR bacterial infection is associated with worse clinical outcomes in patients with bronchiectasis, including a longer duration of mechanical ventilation, prolonged ICU and hospital stays, and increased mortality.

Bacteria colonization increase the risk of mortality in bronchiectasis and is incorporated into BSI as a clinical prediction tool [6]. The mortality rates associated with colonizing bacterial species varied significantly in an international multicenter study, which derives and validates BSI score and reported that *P. aeruginosa* (21.2%) and MRSA (62.5%) infections exhibited the highest mortality rates among MDR bacterial species [6]. Although the culture results of that study revealed that only 8 of 1,310 patients with bronchiectasis had MRSA infection, the high mortality rate was an alarming finding that warranted further exploration [6]. Studies have reported that MDR bacterial infections involving *P. aeruginosa*, ESBL *Enterobacter*, and MRSA were independently associated with an increased risk of 30-day mortality in the general population [9, 30]. However, the effects of MDR bacterial species on the outcomes of bronchiectasis remain unclear. The findings of the present study indicate that in-hospital mortality rates were not significantly different among the MDR subgroups. However, among these MDR subgroups, the MDR-*Pseudomonas* subgroup exhibited the highest 3-year respiratory failure rates, whereas the ESBL-*KP* subgroup exhibited the highest 3-year mortality rates.

Several risk factors have been reported to be associated with MDR infection in hospitalized patients, including previous exposure to antibiotics, use of invasive catheterization devices, mechanical ventilation, and hospital admission (particularly admission to an ICU) [31]. In patients with bronchiectasis, several risk factors for MDR bacterial infection during exacerbations have been identified, including hospitalization within the preceding year, chronic kidney disease, and previous MDR isolation [11]. In the present study, the MDR group exhibited lower FEV1 levels and BMI scores, higher BACI scores, a higher rate of previous exacerbation, and an increased use of antibiotics relative to the control group; these findings indicate the presence of more comorbid diseases and a higher disease severity. Age, hematological malignancy, and acute kidney injury were identified as risk factors for in-hospital mortality. Age, male sex, per-unit decrease in BMI score, acute kidney injury, hemodialysis, systemic steroid use, and BACI score were identified as risk factors for 3-year mortality. The risk factors for poor clinical outcomes due to MDR bacterial infection include inappropriate administration of initial antibiotic therapy, underlying comorbidities, and poor immunity [10]. Thus, clinicians should identify patients at risk of MDR bacterial infection from the onset of infection.

Decolonization of MDR bacteria has been proposed to reduce subsequent infection risk and improve clinical outcomes [32]. For carbapenem-resistant enterobacteriaceae colonization in digestive tract, oral antibiotic decolonization has been reported to be effective in reducing mortality [33, 34]. However, the routine decolonization of other MDR bacteria (MDR-gram-negative bacteria, carbapenem-resistant AB, and third-generation cephalosporin-resistant Enterobacteriaceae) is not recommended [32]. In the present study, MDR nonconversion contributed to a higher risk of 3-year mortality in the MDR group than in the control group. Although MDR conversion was associated with a lower risk of 3-year mortality relative to MDR nonconversion, MDR conversion was still an independent risk factor for 3-year mortality compared to the control group. The present study provides evidence of the risk of MDR nonconversion in patients with bronchiectasis. Nevertheless, further prospective clinical research is required to assess the effects of various interventions on patients with both bronchiectasis and MDR bacterial infection.

The prevalence of MDR bacterial infection varies across countries [7, 35]. In numerous countries, the trends in the prevalence of MDR bacterial infection may increase or remain stable, depending on the status of endemic infection or ongoing regional spread. In Asia, the prevalence of MDR bacterial infection is increasing in numerous countries, and the burden of antimicrobial drug resistance is greater in this region than in Western countries [7]. A decrease in the incidence of MDR bacterial infection has occasionally been observed after the implementation of surveillance programs and infection control interventions. In the United States, the incidence of MDR bacterial infection involving the *P. aeruginosa*, MRSA, and carbapenem-resistant *Acinetobacter* species decreased from 2012 to 2017 [24]. This study found that regional differences in MDR bacteria proportion of bronchiectasis existed in the institutes of CGMH and over ten years, the number of patients with bronchiectasis who developed MDR bacteria was increasing. The increasing trend of MDR bacterial infection highlights the crucial role that infection-control measures play in health-care settings in reducing the prevalence of resistant bacteria.

The present study has several limitations. First, although the data retrieved from the CGRD comprised data from medical centers and regional hospitals, such data may differ from those of other hospitals of Taiwan. Second, we could not retrieve several parameters from the CGRD, and BSI scores could not be calculated to stratify our results by disease severity. However, we demonstrated that BACI scores can be used to predict mortality in CGRD [15]. Third, previous exacerbation rates, lung function and BMI were not included in propensity score matching. We list age, gender, comorbidities and BACI score as basis for propensity score matching because BACI score has been validated to predict prognosis in bronchiectasis [21]. In a European study, the BACI predicted 5-year mortality rate, hospital admissions, exacerbations, and health-related quality of life [21]. Our previous study has provided evidence that BACI could be used to accurately stratified the risk of hospital and 1-year follow-up mortality in CGRD [15]. Although we did not include previous exacerbation rates in propensity score matching, exacerbation rates were adjusted in the univariate and multivariate analysis of 3-year mortality when evaluating the effect of MDR bacteria on the outcome. Because there were some missing data in lung function and BMI, we did not include these two parameters in propensity score matching. Fourth, the present study adopted a retrospective observation design and was based on a database of real-world practice. The clinicians may choose different regimens for the treatment of MDR bacteria. Therefore, treatment selection bias may exist when evaluating the outcomes of MDR infection and future prospective study with standard protocol is needed.

## Conclusions

MDR bacteria were identified in a proportion of patients with bronchiectasis and were revealed to be independently associated with an increased risk of in-hospital and 3-year mortality. In the bronchiectasis cohort of the present study, MDR-*AB* was the most frequently isolated MDR bacteria, followed by MRSA, ESBL-KP, MDR-*Pseudomonas*, and ESBL-*E. coli*. In addition to MDR bacterial infection, we identified several independent risk factors for in-hospital and 3-year mortality. Given our findings, we recommend that clinicians identify patients at risk of MDR bacterial infection and follow the principle of antimicrobial stewardship to prevent the emergence of resistant bacteria among patients with bronchiectasis.

### Electronic supplementary material

Below is the link to the electronic supplementary material.

**Supplementary Material 1: sTable 1.** The standard criteria of antibiotics sensitivity by disc diffusion method in CGMH; **sTable 2.** Demographics and Clinical Characteristics before and after propensity score matching

## Data Availability

The data are not publicly available due to ethical restrictions and regulations of the Institutional Review Board of Chang Gung Memorial Hospital.

## Abbreviations

AB: Acinetobacter baumannii
BACI: Bronchiectasis Aetiology Comorbidity Index
BMI: body mass index
BSI: bronchiectasis severity index
CGMH: Chang Gung Memorial Hospital
CGRD: Chang Gung Research Database
COPD: chronic obstructive pulmonary disease
ICD-9-CM: International Classification of Diseases, 9th Clinical Modification
ICU: intensive care units
E. coli: Escherichia coli
ESBL: extended-spectrum-beta-lactamases
KP: Klebsiella pneumoniae
MDR: multidrug-resistant
MRSA: methicillin-resistant *Staphylococcus aureus*
