# Supplementary material for: Epidemiology and outcomes of multidrug-resistant bacterial infection in non-cystic fibrosis bronchiectasis
Source: Ann Clin Microbiol Antimicrob. 2024 Feb 13;23:15. doi: 10.1186/s12941-024-00675-6 (PMC10865664; doi:10.1186/s12941-024-00675-6)
Supplement: Supplementary file 1 — Supplementary Material 1: sTable 1. The standard criteria of antibiotics sensitivity by disc diffusion method in CGMH; sTable 2. Demographics and Clinical Characteristics before and after propensity score matching [file 12941_2024_675_MOESM1_ESM.docx]

**Supplementary Information**

**Epidemiology and outcomes of multidrug-resistant bacteria infection in non-cystic fibrosis bronchiectasis**

***Chih-Hao Chang ^1, 2, 3^, Chiung-Hsin Chang ^2, 3^, Shih-Hao Huang ^1, 2, 3^, Chung-Shu Lee ^1, 2, 3^, Po-Chuan Ko ^4^, Chun-Yu Lin ^2, 3^, Meng-Heng Hsieh ^2, 3^, Yu-Tung Huang ^4^, Horng-Chyuan Lin ^2, 3^, Li-Fu Li ^2, 3, 5^, Fu-Tsai Chung ^1, 2, 3^, Chun-Hua Wang ^2, 3^ and Hung-Yu Huang ^1, 2, 3^***

*Affiliations*

*^1^ Department of Thoracic Medicine, New Taipei City Municipal TuCheng Hospital, Chang Gung Medical Foundation, New Taipei City, Taiwan*

*^2^ College of Medicine, Chang Gung University, Taoyuan, Taiwan*

*^3^ Department of Thoracic Medicine, Chang Gung Memorial Hospital, Taipei, Taiwan.*

*^4^ Center for Big Data Analytics and Statistics, Chang Gung Memorial Hospital, Taoyuan, Taiwan.*

*^5^ Division of Pulmonary and Critical Care Medicine, Department of Internal Medicine, Chang Gung Memorial Hospital, Keelung, Taiwan*

sTable 1 The standard criteria of antibiotics sensitivity by disc diffusion method in CGMH

Note: CGMH, Chang Gung Memorial Hospital

sTable 2 Demographics and Clinical Characteristics before and after propensity score matching

| Characteristic | Before Matching | |  | Matched | |  |
| --- | --- | --- | --- | --- | --- | --- |
|  | Control group | MDR group | P value | Control group | MDR group | P value |
|  | N= 7359 | N=554 |  | N= 1108 | N= 554 |  |
| Age | 67.6 ± 14.2 | 75.1 ± 12.7 | <.0001 | 75.0 ± 11.7 | 75.1± 12.6 | 0.967 |
| Gender, female | 4067 (55.3%) | 228 (41.2%) | <.0001 | 455 (41.1%) | 228 (41.2%) | 0.971 |
| BACI index | 7.1 ± 6.1 | 11.3 ± 6.4 | <.0001 | 11 ± 6.8 | 11.3 ± 6.4 | 0.358 |
| Comorbidity |  |  |  |  |  |  |
| Solid tumor | 495 (6.7%) | 64 (11.6%) | <.0001 | 126 (11.4%) | 64 (11.55%) | 0.913 |
| Hematological malignancy | 280 (3.8%) | 25 (4.5%) | 0.404 | 56 (5.1%) | 25 (4.5%) | 0.628 |
| COPD | 3495 (47.5%) | 365 (65.9%) | <.0001 | 737 (66.5%) | 365 (65.9%) | 0.797 |
| Liver disease | 1509 (20.5%) | 143 (25.8%) | 0.003 | 308 (27.8%) | 143 (25.8%) | 0.390 |
| Connective tissue disease | 337 (4.6%) | 39 (7.0%) | 0.0087 | 84 (7.6%) | 39 (7.0%) | 0.691 |
| Diabetes | 1603 (21.8%) | 226 (40.8%) | <.0001 | 452 (40.8%) | 226 (40.8%) | 1.000 |
| Asthma | 2316 (31.5%) | 256 (46.2%) | <.0001 | 500 (45.1%) | 256 (46.2%) | 0.676 |
| Ischemic heart disease | 1309 (17.8%) | 151 (27.3%) | <.0001 | 348 (31.4%) | 151 (27.3%) | 0.081 |
| Cerebrovascular disease | 1209 (16.4%) | 206 (37.2%) | <.0001 | 418 (37.7%) | 206 (37.2%) | 0.829 |
| Cardiovascular disease | 2083 (28.3%) | 285 (51.4%) | <.0001 | 586 (52.9%) | 285 (51.4%) | 0.578 |
| Chronic renal disease | 1069 (14.5%) | 176 (31.8%) | <.0001 | 363 (32.8%) | 176 (31.8%) | 0.683 |
| GERD | 1266 (17.2%) | 176 (31.8%) | <.0001 | 359 (32.4%) | 176 (31.8%) | 0.795 |
| Osteoporosis | 1111 (15.1%) | 141 (25.5%) | <.0001 | 272 (24.6%) | 141 (25.5%) | 0.688 |

Note: BACI, bronchiectasis aetiology comorbidity index; COPD, chronic obstruction pulmonary disease; GERD, Gastroesophageal reflux disease; MDR: multidrug- resistant
